# Machine learning models for prediction of HF and CKD development in early-stage type 2 diabetes patients

**DOI:** 10.1038/s41598-022-24562-2

**Published:** 2022-11-21

**Authors:** Eiichiro Kanda, Atsushi Suzuki, Masaki Makino, Hiroo Tsubota, Satomi Kanemata, Koichi Shirakawa, Toshitaka Yajima

**Affiliations:** 1 Medical Science, Kawasaki Medical University, Okayama, Japan; 2 Department of Endocrinology, Diabetes and Metabolism, Fujita Health University, Toyoake, Aichi Japan; 3 AstraZeneca K.K., Osaka, Japan; 4 Ono Pharmaceutical Co., Ltd., Osaka, Japan

**Keywords:** Computational science, Scientific data, Cardiovascular biology, Experimental models of disease, Kidney diseases, Type 2 diabetes

## Abstract

Chronic kidney disease (CKD) and heart failure (HF) are the first and most frequent comorbidities associated with mortality risks in early-stage type 2 diabetes mellitus (T2DM). However, efficient screening and risk assessment strategies for identifying T2DM patients at high risk of developing CKD and/or HF (CKD/HF) remains to be established. This study aimed to generate a novel machine learning (ML) model to predict the risk of developing CKD/HF in early-stage T2DM patients. The models were derived from a retrospective cohort of 217,054 T2DM patients without a history of cardiovascular and renal diseases extracted from a Japanese claims database. Among algorithms used for the ML, extreme gradient boosting exhibited the best performance for CKD/HF diagnosis and hospitalization after internal validation and was further validated using another dataset including 16,822 patients. In the external validation, 5-years prediction area under the receiver operating characteristic curves for CKD/HF diagnosis and hospitalization were 0.718 and 0.837, respectively. In Kaplan–Meier curves analysis, patients predicted to be at high risk showed significant increase in CKD/HF diagnosis and hospitalization compared with those at low risk. Thus, the developed model predicted the risk of developing CKD/HF in T2DM patients with reasonable probability in the external validation cohort. Clinical approach identifying T2DM at high risk of developing CKD/HF using ML models may contribute to improved prognosis by promoting early diagnosis and intervention.

## Introduction

Globally, an estimated 537 million people are affected by type 2 diabetes mellitus (T2DM), which is an established risk factor for chronic kidney disease (CKD) and cardiovascular diseases (CVDs), including heart failure (HF)^1–5^. It is reported that diabetic kidney disease (DKD) develops in approximately 40% of diabetic patients, representing as the leading cause of CKD and end-stage renal disease worldwide^3,6–8^. CVDs affect approximately 32.2% of all people with T2DM^9^, and patients with diabetes are known to be at a two-fold increased risk for developing HF^10,11^ and CVD.

In Japan, as of 2018, approximately 10 million individuals had diabetes, about half of whom were over 65 years old^1,12^. The Japan Diabetes compREhensive database project based on an Advanced electronic Medical record System (J-DREAMS) study showed that 35.4% had CKD and 22.1% had CVD, with prevalence increasing with age and disease duration^13^, based on the analysis of 10,151 Japanese patients with T2DM between 2017 and 2019. Previous studies have identified that manifestation of CKD and/or HF (CKD/HF) is associated with increased mortality compared to patients with T2DM without a history of CKD or CVDs. Birkeland et al. showed CKD/HF to be the most frequent initial manifestations in patients with early stages of T2DM ^14^ by analyzing a database of 1,177,896 patients from six different countries, including Japan. Given that the early and improved diagnosis of these conditions can improve outcomes^15^, a comprehensive treatment approach to control CKD and HF in patients with early stages T2DM is desirable^3,16^. However, current evidence suggests that CKD and HF are not optimally diagnosed in patients with T2DM , leading to higher rates of disease progression and poor prognosis^17–20^.

Multiple prediction models using machine learning (ML) techniques have been built over the last few years for risk assessment accounting for diabetes severity, complications, hospitalizations, disease progression, and adverse outcomes^21–29^. While algorithms developed by ML can analyze complex interactions using expansive data to improve discrimination, learning patterns, and decision rules^30,31^, currently, there are no ML algorithms for the prediction of developing CKD/HF among patients in the early stages of T2DM before CKD/HF manifests.

This study aimed to build a model that could predict the risk of developing CKD/HF among patients with T2DM without a history of CKD or CVDs using statistical methods and ML techniques. Utilizing the prediction model would enable the early identification of patients with T2DM who are at high risk of developing CKD/HF; ultimately, the optimization of treatment interventions based on the risk assessment may improve the prognosis of these patients.

## Methods

### Ethical statement

This study was performed in accordance with ethical principles that are consistent with the Declaration of Helsinki, International Council for Harmonisation Good Clinical Practice, Good Pharmacoepidemiology Practice, and the applicable legislation on noninterventional studies and/or observational studies. In this study, we used two anonymized, publicly available commercial databases obtained from Japan Medical Data Vision Co., Ltd. and Real World Data Co., Ltd. Institutional review board approvals were not needed because the current study only involves secondary analysis of de-identified, anonymized data. In Japan, ethical approval and informed consent do not apply to the use of de-identified secondary data according to the Japanese Ethical Guidelines for Medical and Health Research Involving Human Subjects.

### Data source and study population

Data were collated from the Japan Medical Data Vision (MDV) database from April 2008 to September 2018 (Supplementary Table S1). The database contains administrative claims and laboratory data from 376 Japanese diagnosis procedure combination (DPC) hospitals, representing 21.7% of the 1,730 DPC hospitals in Japan, covering approximately 20 million patients in Japan^32^. In this database, data were classified using the International Classification of Diseases, 10th Revision (ICD-10) diagnosis codes; disease names are coded using Japanese-specific disease codes, and procedures and drug prescriptions and administration are coded using Japanese-specific receipt codes^32^.

Patients aged ≥ 18 years with a diagnosis of T2DM receiving antidiabetic treatment and with an 18-months run-in interval prior to the index date were included. Patients with a diagnosis of type 1 diabetes mellitus at any time in the database, with a diagnosis of gestational diabetes at any time in the database, or with medical history of CVDs or CKD prior to the index date were excluded. Supplementary Table S2 shows the list of Anatomical Therapeutic Chemical (ATC), ICD-10, and procedure codes used for the inclusion and exclusion criteria.

The index date was defined as the date when the first oral medication for T2DM was prescribed after the diagnosis of T2DM and must be more than 18 months after the starting date of observation (lookback period). The lookback period was set at a minimum of 18 months before the index date to secure a sufficiently long pre-index period to enable proper collection of information on patient background characteristics and avoid information bias owing to seasonal fluctuations.

### Outcomes and variables

Risk prediction models were created for the following clinical outcomes: The primary outcomes were (1) diagnosis of CKD/HF in an inpatient or outpatient setting, and (2) hospitalization for CKD/HF or for uncertain reasons such as the maximum healthcare resource usage during admission related to CKD/HF. Secondary outcomes were (1) diagnosis of HF (inpatient or outpatient), (2) diagnosis of CKD (inpatient or outpatient), and (3) hospitalization for HF or for uncertain reasons such that the maximum healthcare resource usage during admission was relating to HF. Lastly, exploratory outcomes were (1) composite major adverse cardiovascular events (MACE)—diagnosis of myocardial infarction (MI), stroke, or in-hospital death related to MI or stroke; (2) composite major adverse renal and cardiovascular events (MARCE); diagnosis of MI, stroke, or hospitalization due to HF; renal outcomes (dialysis and kidney transplant); or in-hospital death related to MI, stroke, or HF; and 3) all in-hospital deaths. Supplementary Table S3 shows the list of ICD-10 and procedure codes used for outcomes.

Variables included patient demographics (age, sex, BMI, frequency of outpatient visit, and frequency of hospitalization), ICD-10 codes, ATC diagnosis of disease codes, and laboratory values derived from the MDV database. Laboratory values were categorized, and patients with measurements were assigned to the categories of normal, below normal, and above normal based on the common criteria for major laboratory parameters in Japan; patients without measurements were classified as having no measurements.

### Model building

The architecture of the model was developed in two separate phases (Supplementary Fig. S1). The first phase included a feasibility assessment of algorithm development and evaluation of variables. The second phase included development and fine-tuning of the full prediction model to finalize and validate the model. In both phases, 80% of the entire analysis dataset was used for model construction and 20% was used for internal validation.

#### Phase I: preliminary model

Data preprocessing included inputting explanatory variables, handling laboratory data, and missing data. As laboratory data were adopted as a continuous variable, outliers were not detected (Step 1). For modeling, 32 models were constructed and evaluated per the method corresponding to eight outcomes and four time points (1, 2, 3, and 5 years after the index date). The preliminary model construction differed from the development of the full prediction model in various aspects, including the random selection of a population of 10,000 individuals with a 1:1 positive-to-negative ratio; laboratory values were not categorized, and missing values were imputed using mean values. The preliminary model was built using random forest and logistic regression methods; and performance of the model was evaluated using area under the receiver operating characteristic curve (AUROC), precision, accuracy, and recall (Step 2).

#### Phase II: full prediction model

Two different techniques (gradient boosting [XGB] and deep learning [multilayer perceptron]) were used for model construction in Phase II by using traditional statistical models (logistic regression and Cox proportional hazards) as comparators. While all positive patients were selected, negative patients were randomly selected to be twice as many as positive patients for model building, resulting in a 1:2 ratio of positive:negative patients. In primary outcomes, the number of explanatory variables used in the analysis was assumed to be 60, with the coefficient of determination adjusted for degree of freedom (R^’2^) as the measure of model fit.

The selection of explanatory variables was first performed by univariate regression analysis using 0.05 as the threshold of the probability of each outcome event. After the selection, 60 variables’ data were extracted using the random forest method with Gini importance, and the data were categorized by quality.

After building models using the XGB and neural networks, hyperparameters were determined using a random search method to increase the accuracy of model-based prediction^33^. Supplementary Tables S4 and S5 show the range of hyperparameters used for model construction. For fine-tuning of the model, 16 categorized laboratory variables (Supplementary Fig. S2) were included in addition to the selected 60 variables. The additional 16 variables were selected based on a factor number determining method of factor analysis (Supplementary Fig. S3). The model was validated by evaluating model performance using AUROC, accuracy, precision, recall, and specificity.

All procedures for model development were implemented using Python 3.9.5. Additionally, SHapley Additive exPlanation (SHAP) analysis was performed for the XGB to identify whether variables with the highest variable importance contributed positively or negatively to the event occurrence^34^ (Step 4).

### External validation

The XGB, which exhibited the best predictive performance in all outcomes, was subjected to external validation using a dataset obtained from Real World Data Co., Ltd. (RWD; Kyoto, Japan). This database contains electronic medical records and claims data consisting of approximately 20 million patients from more than 160 medical institutions across Japan, as of 2020. It includes information of patient characteristics, diagnoses, prescriptions, procedures, and laboratory data for both inpatient and outpatient cares. These data are routinely collected within each individual medical institution and anonymized using identifiers for each patient. We used only DPC data in the RWD database to perform consistent analysis with internal validation.

In this analysis, accuracy of the model was evaluated based on AUROC, precision, recall, and specificity for each outcome. Furthermore, for Kaplan–Meier analysis, patients were divided into high- and low-risk groups based on the best cutoff value determined by the receiver operating characteristic (ROC) curve, obtained as the point on the ROC curve that provides the shortest distance between the arc of the ROC curve and upper-left corner of the unit square (sensitivity = 1, specificity = 0). This point is the optimal cutoff point (threshold) for distinguishing the two groups in survival analysis. The log-rank test was used to compare the two curves.

These external validation analyses were performed independently from the model development to ensure the reliability of the results.

## Results

### Baseline characteristics

**Figure 1:**
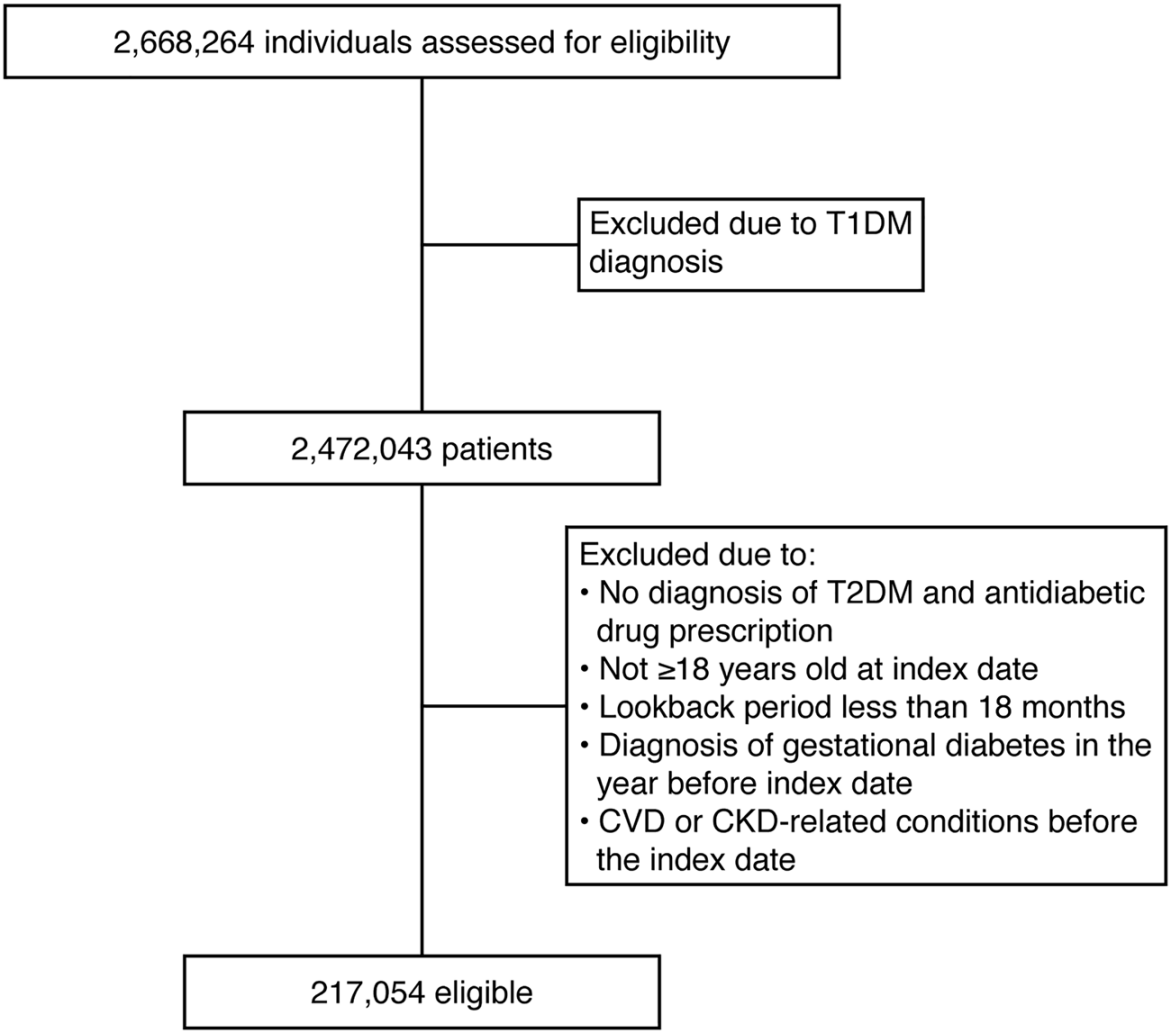
Patient disposition. CKD, chronic kidney disease; CVD, cardiovascular disease; T1DM, type 1 diabetes mellitus; T2DM, type 2 diabetes mellitus.

Of the 2,668,264 individuals identified in the MDV database between April 2008 and September 2018, eligible individuals (n = 217,054) were included in the analysis (Fig. 1).

For external validation, we selected 16,822 patients from the RWD database based on the inclusion and exclusion criteria (Supplementary Fig. S4). The baseline demographic characteristics of the study population are presented in Table 1.

**Table 1:** Baseline characteristics.

Characteristic	Number of patients (N)	Mean (SD)
Age, years	217,054	67.7 (12.99)
Male, %	125,561	57.8
BMI below 18.5 kg/m^2^, %	10,515	4.8
BMI 18.5–24.9 kg/m^2^, %	68,086	31.4
BMI 25.0–29.9 kg/m^2^, %	33,220	15.3
BMI 30.0–34.9 kg/m^2^, %	9,146	4.2
BMI 35.0–39.9 kg/m^2^, %	2,202	1
BMI above 40 kg/m^2^, %	799	0.37
White blood cell count, /μL	30,703	7,880.18 (6,580.72)
Red blood cell count, ×10,000/μL	30,705	423.36 (73.63)
Hemoglobin, g/Dl	30,705	12.89 (2.25)
Hematocrit, %	30,704	38.39 (6.28)
Platelet count, ×10,000/μL	30,703	22.14 (8.96)
Neutrophils, %	17,016	67 (13.63)
Eosinophils, %	18,050	2.28 (2.77)
Glucose, mg/dL	25,727	184.04 (100.81)
Glycated hemogloblin A1c, %	12,325	7.09 (1.68)
Triglycerides, mg/dL	18,140	154.11 (196.51)
Cholesterol, mg/dL	18,679	185.35 (47.69)
HDL-C, mg/dL	14,711	52.73 (16.75)
LDL-C, mg/dL	13,259	111.3 (36.37)
GOT, U/L	31,324	36.85 (109.07)
Frequency of outpatient visits, times/day	217,054	0.0127 (0.0196)
Frequency of hospitalization, times/day	217,054	0.0007 (0.0014)

*BMI* body mass index, *GOT* glutamic oxaloacetic transaminase, *HDL-C* high-density lipoprotein cholesterol, *LDL-C* low-density lipoprotein cholesterol, *SD* standard deviation.

### Preliminary modeling performance

The AUROCs for CKD/HF diagnosis at the four time points were 0.640–0.727 with logistic regression and 0.678–0.779 with random forest analysis (Supplementary Table S6), which indicated reasonable performance of models based on MDV-derived data.

### Selection of explanatory variables

Of approximately 1000 variables analyzed, the top 60 were chosen based on variable importance evaluated by the Gini coefficient (Supplementary Fig. S5 and S6) to develop the full prediction model. The top-ranking variables were age, frequency of outpatient visits, and frequency of hospitalizations.

### Initial full prediction model performance

**Figure 2:**
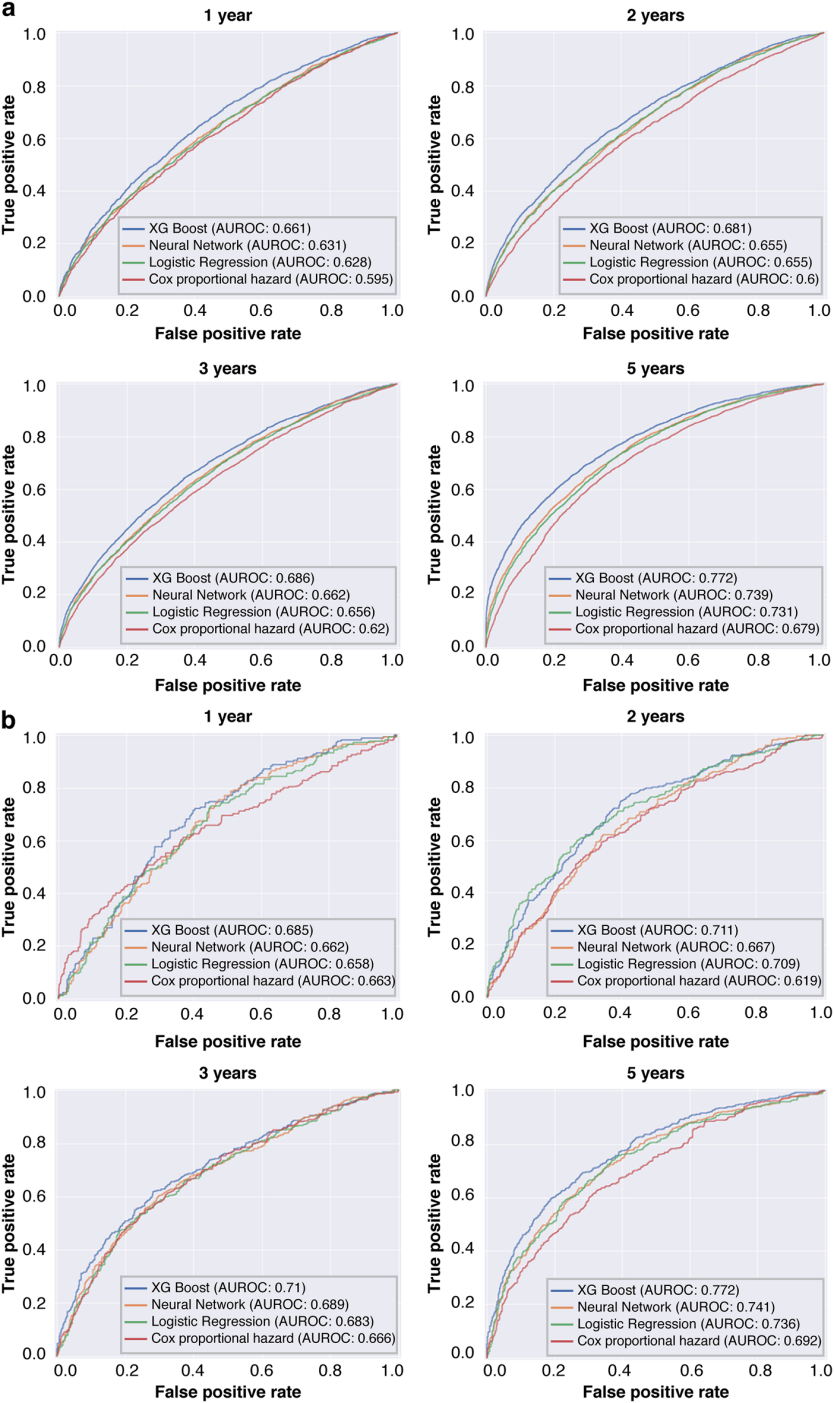
Performance of full prediction models with top 60 variables. (**a**) ROC curves of different types of models for predicting the risk of diagnosis of CKD/HF and (**b**) ROC curves of different types of models for predicting the risk of hospitalizations for CKD/HF. *AUROC* area under the receiver operating characteristic curve, *CKD* chronic kidney disease, *HF* heart failure, *ROC* receiver operating characteristic.

The model built using the top 60 variables showed similar performance compared with the preliminary models constructed using all variables. Among four (two ML and two statistical) methods, the XGB technique showed a higher AUROC than the other techniques in all outcomes and time points examined; the AUROC ranged between 0.661 and 0.772 for CKD/HF diagnosis and between 0.685 and 0.772 for hospitalization for CKD/HF among different time points (Fig. 2).

### Performance of full prediction model after fine-tuning

When AUROCs of XGB were compared between patients with available laboratory data and those without any laboratory data, the AUROCs increased by 0.015–0.105 for CKD/HF diagnosis and by 0.045–0.139 for CKD/HF hospitalization (Supplementary Table S7). Therefore, for model fine-tuning, 16 laboratory variables were included in the models.

**Figure 3:**
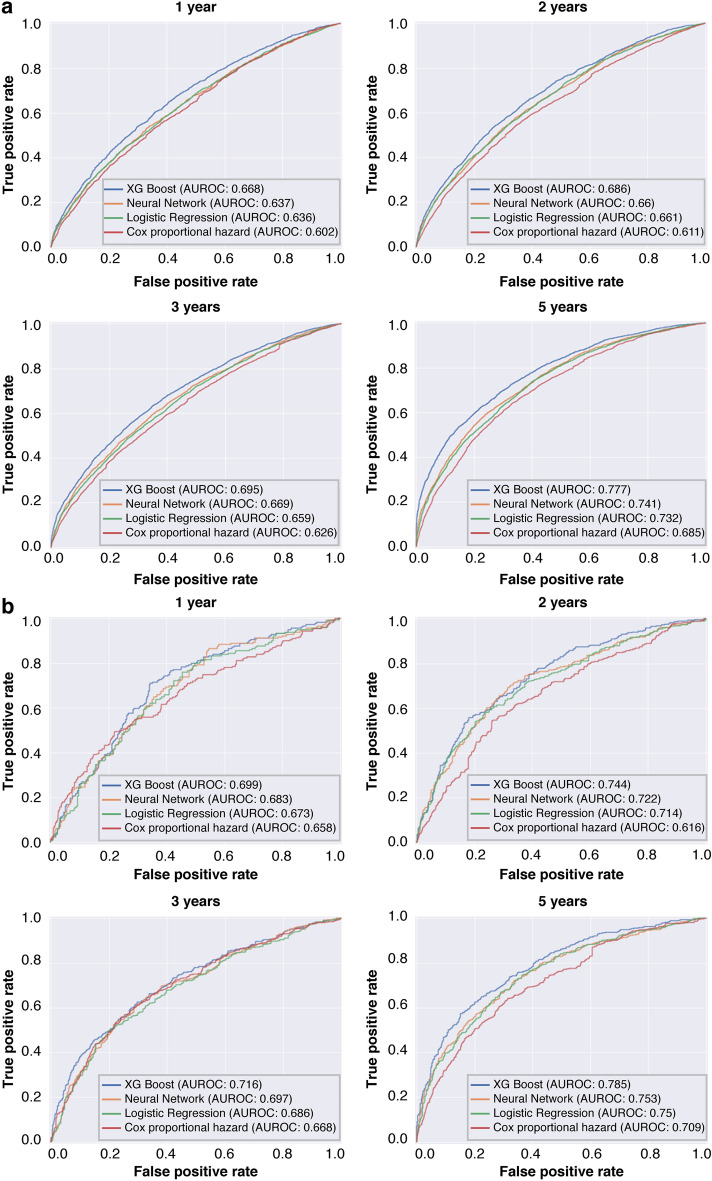
Performance of full prediction models with top 60 variables and 16 laboratory values. (**a**) Diagnosis of CKD/HF and (**b**) hospitalizations for CKD/HF. *AUROC* area under the receiver operating characteristic curve, *CKD* chronic kidney disease, *HF* heart failure.

On comparing all evaluated techniques, the XGB technique resulted in higher AUROCs (Fig. 3). The 5-years prediction AUROC results for primary outcomes using the XGB full predictive model after fine-tuning were 0.777 for the diagnosis of CKD/HF and 0.785 for hospitalization for CKD/HF (Table 2). For secondary and exploratory outcomes, the AUROCs during year 1 to year 5, ranged from 0.728 to 0.799 for HF, from 0.681 to 0.770 for CKD, from 0.754 to 0.809 for HF hospitalization, from 0.724 to 0.792 for MACE composite, from 0.687 to 0.791 for MARCE composite, and from 0.904 to 0.918 for death, with the lowest AUROC observed for year 1 and the highest for year 5 (Table 2).

**Table 2:** AUROCs for the XGB model using top 60 variables and 16 laboratory values.

Outcomes	Year			
	1	2	3	5
Diagnosis of CKD/HF	0.668	0.686	0.695	0.777
Hospitalization for CKD/HF	0.699	0.744	0.716	0.785
Diagnosis of CKD	0.681	0.671	0.691	0.770
Diagnosis of HF	0.728	0.743	0.740	0.799
HF hospitalization	0.754	0.770	0.741	0.809
Death	0.904	0.906	0.902	0.918
MACE composite	0.724	0.744	0.732	0.792
MARCE composite	0.687	0.720	0.729	0.791

*AUROC* area under the receiver operating characteristic curve, *CKD* chronic kidney disease, *HF* heart failure, *MACE* major adverse cardiovascular event, *MARCE* major adverse renal and cardiovascular events.

**Figure 4:**
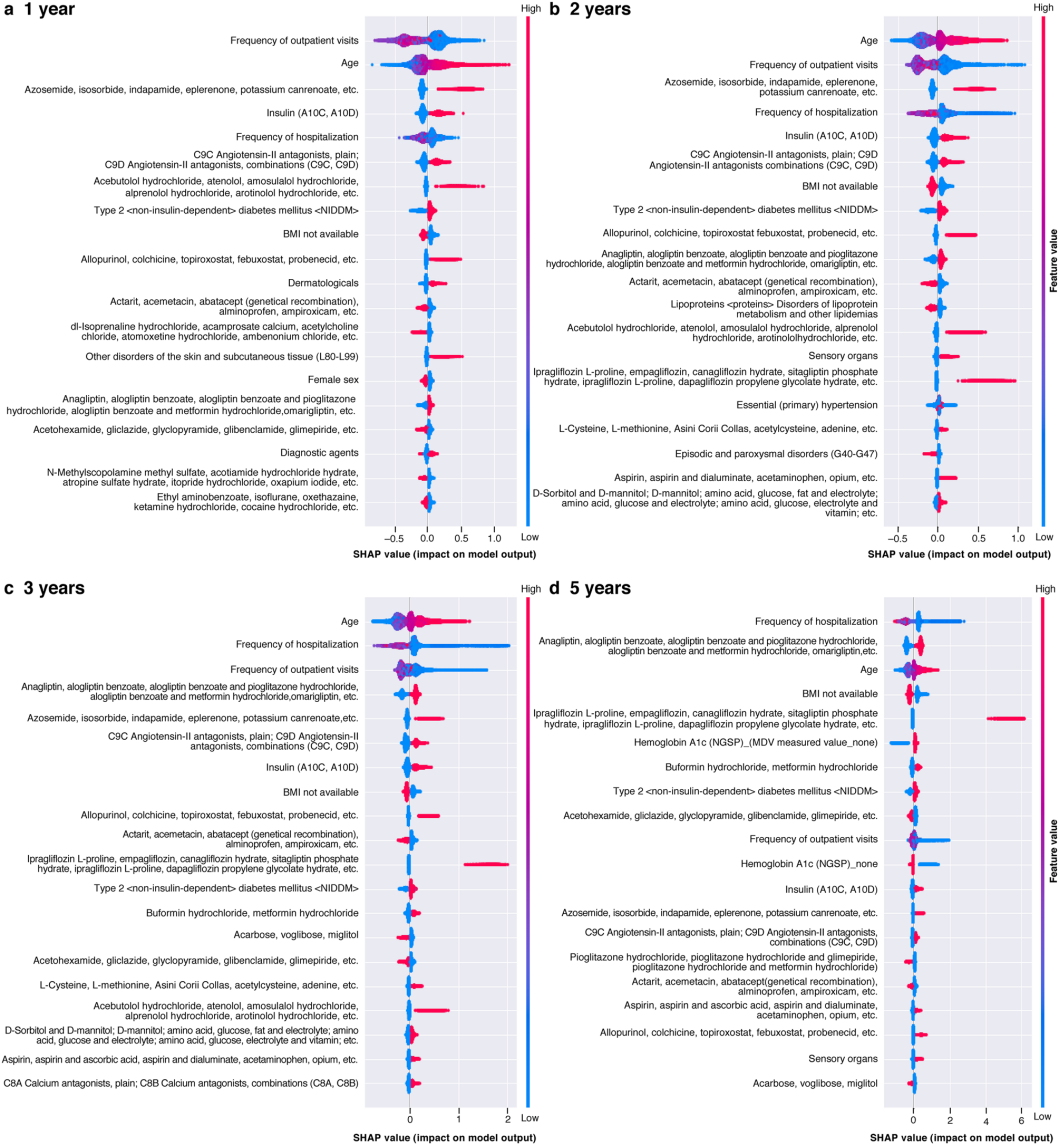
SHAP summary plot in XGB model with 60 variables and 16 laboratory values. (**a**)–(**d**) Diagnosis of CKD/HF. *BMI* body mass index, *CKD* chronic kidney disease, *HF* heart failure, *NGSP* National Glycohemoglobin Standardization Program, *SHAP* SHapley Additive exPlanation.

**Figure 5:**
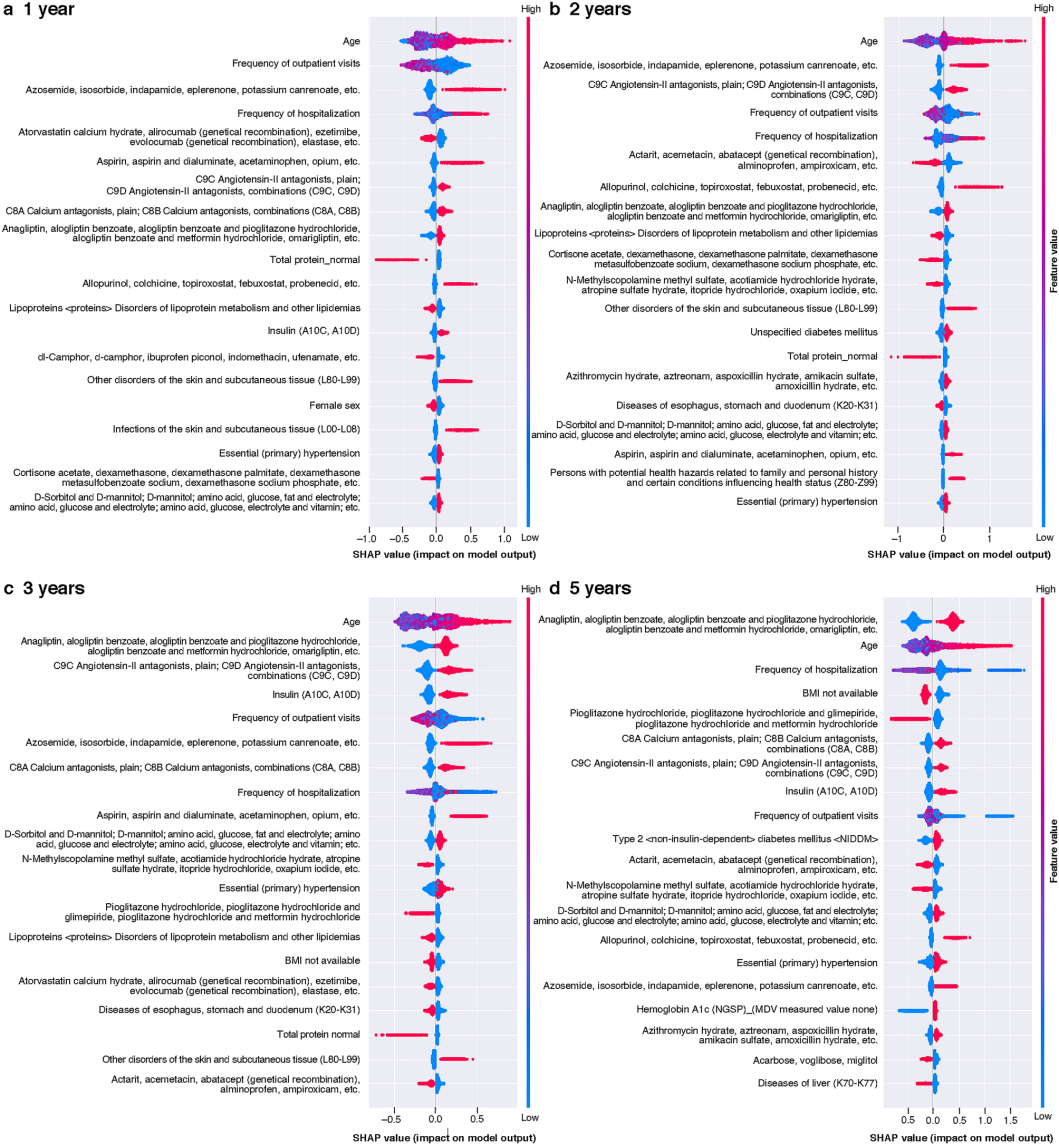
SHAP summary plot in XGB model with 60 variables and 16 laboratory values. (**a**)–(**d**) Hospitalizations for CKD/HF. *BMI* body mass index, *CKD* chronic kidney disease, *HF* heart failure, *NGSP* National Glycohemoglobin Standardization Program, *SHAP* SHapley Additive exPlanation.

SHAP analysis was performed on the XGB. The effect and importance of each variable are described in a SHAP summary plot, showing a higher feature value for the frequency of outpatients visits, age, frequency of hospitalization, and loop diuretic variables (Figs. 4, 5).

The estimated risk for CKD/HF diagnosis tended to increase with age and use of medications, such as loop diuretics and insulin. Below-average frequency of outpatient visits also had an effect, increasing the diagnosis of CKD/HF. A similar trend was observed for CKD/HF hospitalizations.

### External validation

**Figure 6:**
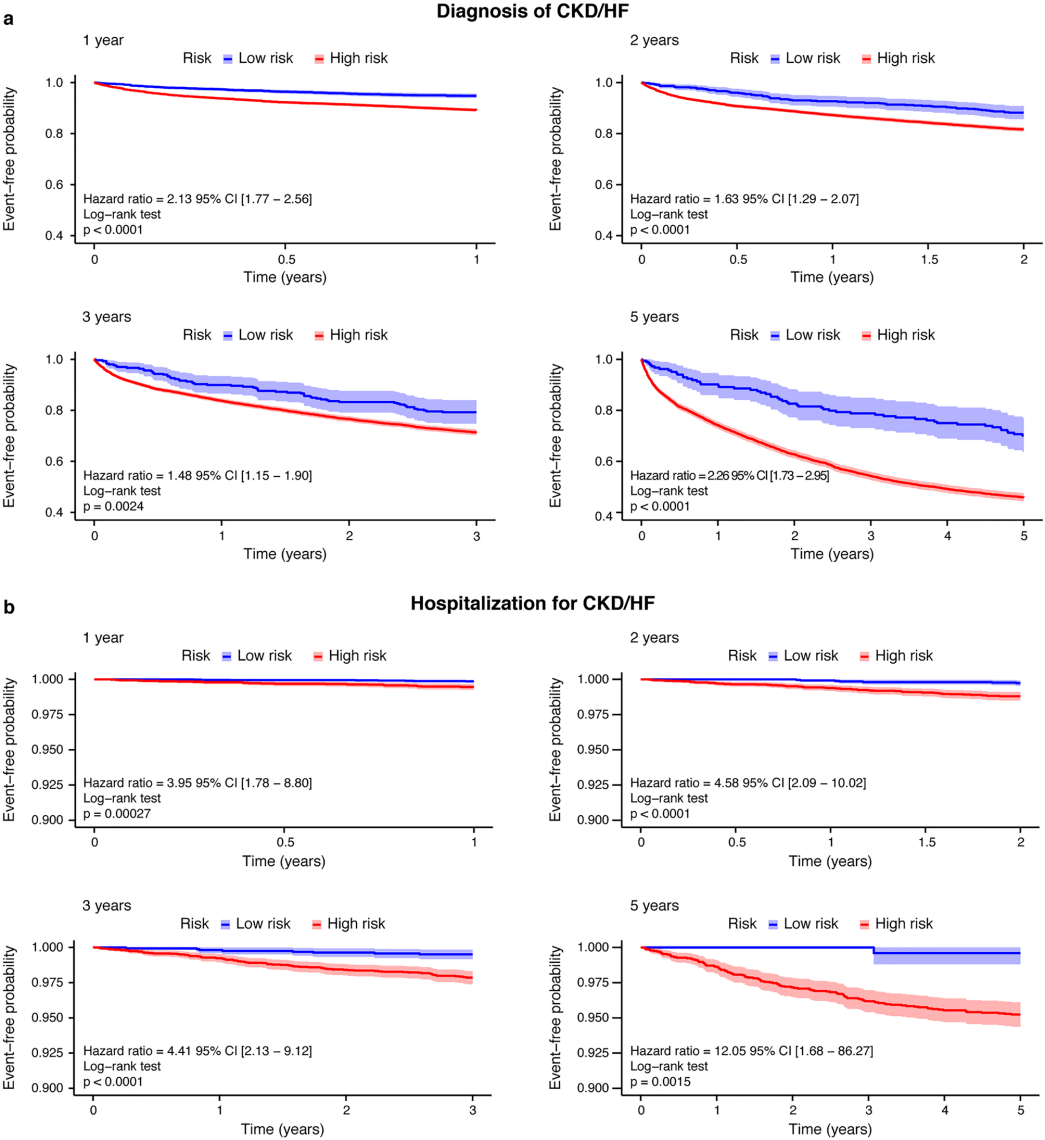
Kaplan–Meier plots of the high-risk and low-risk groups based on risk predictions for primary outcomes in the external validation set. (**a**) Diagnosis of CKD/HF and (**b**) hospitalizations for CKD/HF. *CKD* chronic kidney disease, *HF* heart failure.

The prediction performance is shown in Supplementary Table S8. The AUROC values for the diagnosis of CKD/HF, hospitalization for CKD/HF, diagnosis of CKD, diagnosis of HF, HF hospitalization, all-cause death, MACE, and MARCE in 5 years were 0.718, 0837, 0.690, 0.752, 0.898, 0.869, 0.743, and 0.695, respectively. The Kaplan–Meier curves for the high-risk and low-risk groups identified based on the best cutoff values showed a significantly higher incidence in the high-risk group for all outcomes (Fig. 6, Supplementary Fig. S7, *p* < 0.005; log-rank test).

## Discussion

Using standard statistical methods and different ML techniques, we developed four different predictive models to evaluate the risk of CKD/HF in T2DM patients without a history of CKD or CVD. To our knowledge, this is the first study to predict the risk of developing CKD/HF among patients in the early stages of T2DM, employing different ML methods. Furthermore, SHAP analyses identified several novel factors associated with the development of CKD/HF, including frequencies of outpatient visit and hospitalization. Our model was validated using a dataset different from the one used for model development. The AUROC values were generally maintained; the prediction accuracy was also supported with Kaplan–Meier analysis, which demonstrated statistically significant separation of low-risk and high-risk patients. As one of the advantages of using the ML technique, our model included variables usually not considered during clinical assessment by physicians, and it could serve as a complementary tool to identify high-risk patients, independent of the physician’s experience. Furthermore, visualizing patient risk could facilitate effective communication between physicians, medical staff, patients, and their families, ultimately supporting shared decision-making for optimal medical treatment.

The performance of the current model was similar to that of previously reported models using ML techniques, such as the WATCH-DM risk score to predict HF at 5 years (AUROC, 0.70)^28^, the prediction model for DKD progression at 6 months by Fujita Health University (AUROC, 0.74)^25^, the KidneyIntelX risk score to predict the aggravation of DKD at 5 years (AUROC, 0.77)^35^, and the DKD risk prediction model in patients with T2DM and normo-albuminuria at 3 years (AUROC, 0.815)^36^. The model using XGB exhibited a higher predictive performance for primary outcomes in these studies^31,37–40^, consistent with that in the present study.

Unlike conventional studies that predict diabetes or related complications through known risk factors^41^, we included more than 1,000 variables for our evaluation and chose explanatory variables that had high impact on the prediction of the outcomes. This approach is expected to help in better understanding factors other than clinical presentations associated with the development and progression of CKD and HF in patients with T2DM. We performed SHAP analysis for the most substantial data features contributing to the performance of the XGB model, which enables the interpretation of complex model outputs^34^. The goal of the SHAP analysis is to explain an ML model’s prediction by calculating the contribution of each feature to the prediction. The Shapley value is a method for showing the relative impact of each feature (or variable) that is being measured on the eventual output of the ML model by comparing the relative effect of the inputs against the average. Results revealed that age, frequency of outpatient visits, hospitalization, and loop diuretic use were associated with a high feature value. As for age and loop diuretic use, many patients had a positive distribution of SHAP, suggesting greater age and loop diuretic use as risk factors of events studied in our analyses. Generally, loop diuretics are used for patients with hypertension or volume overload, which is often caused by cardiac or renal dysfunction. The use of this class of medication may have further impacted renal function. Interestingly, below-average frequency of outpatient visits or hospitalizations was associated with the higher risk of CKD/HF diagnosis and hospitalization. This may suggest that a lack of medical attention by healthcare professionals or low treatment adherence of patients hindered the early detection of CKD or HF and that continuous medical care is important for patients with T2DM even if they are in the early stages of the disease.

A relevant factor in building a clinically meaningful ML model is to use training data that accurately represent the population of interest^42^. Poor data representation portrays a potential demographic bias in the development of ML models^42^. In our study, clinical information was extracted from an administrative claims database that covers approximately 15% of the Japanese population. Demographic characteristics, including the age and sex distributions of these patients, are known to be very similar to those of national statistics in Japan. In our study, the mean values for some of our cohort’s laboratory variables, such as glycated hemoglobin and low-density and high-density lipoprotein cholesterol, were similar to those observed in a previous extensive study that included two cohorts of Japanese patients with diabetes from 2004 and 2014^12^. Another crucial step to assess the clinical applicability, as well as the quality of a prediction model, is external validation^43,44^. In the present study, our model maintained AUROCs in the external validation. Additionally, we evaluated model performance by comparing the high-risk and low-risk groups identified using the best cutoff values. In the 5-year follow-up, the high-risk group exhibited a higher incidence of all outcomes. These validation results indicate the usefulness of our model as a support tool for a wide range of clinicians in the management of patients with T2DM.

Patients with T2DM are at high risk for CKD and HF, and these complications have a significant impact on their prognosis^14,45^. However, there are not enough preventive measures nor early interventions implemented for CKD and HF in patients with T2DM, and one possible cause could be a difficulty in identifying patients at a high risk of these complications. Phenotypic and pathophysiological heterogeneity in HF is well recognized, and its initial clinical presentations are not necessarily specific to HF. In contrast, CKD can be diagnosed using the estimated glomerular filtration rate value or urinalysis data; however, patients do not show significant symptoms or signs in the early stages of the disease, which can delay the testing of renal function. Our model may contribute to increased awareness of the importance of screening for CKD and HF among clinicians, regardless of the disease duration or severity of T2DM.

The algorithm used in the present study may be applicable to other disease areas, including infectious disease epidemiology, and possibly improve the analysis performance. A huge effort has been made to describe, understand, and predict the spread of infectious diseases at different spatial scales, from personal to regional and global, by using mathematical models^46–49^. One example is COVID-19, and recent studies have reported important factors for the COVID-19 pandemic, including the rate of vaccination for social spreading and ventilation conditions for indoor spreading^47,48^. Highly accurate analysis would be possible by using ML and selecting the most appropriate parameters in mathematical models. In addition, in the present study, we performed survival analyses and prognostic assessments. Using the algorithm used in this process as a variable selection method and using data on medical history as variables in a predictive model for the epidemic period^49^, we would be able to estimate the periodicity of outcome occurrence and contributing factors.

This study has limitations. First, the study’s database included information only from DPC-covered hospitals, a system for secondary care hospitals. Considering that patients with T2DM can visit clinics or hospitals outside of the remit of DPC-covered hospitals for regular follow-up, caution should be exercised while interpreting and generalizing the results of this study. Second, as this study used a secondary database, misclassification of explanatory variables and outcomes may have occurred. Third, the databases used in this study only include in-hospital deaths, and the incidence of death may be different from other types of hospitals, such as clinics. Fourth, some data may have been underestimated because the database comprises information exclusively from a subset of hospitals. Lastly, the MDV database presents a high number of patients with missing laboratory variables.

## Conclusion

We built an ML model that could predict the risk of developing CKD or HF and other associated clinical outcomes in patients with T2DM without a previous record of these conditions. We also showed that this model could identify the patients with poor prognosis as high-risk group. Visualizing patient risk may contribute to interdisciplinary intervention and shared decision-making by facilitating effective communication among physicians, medical staffs, patients, and their families. Furthermore, SHAP analysis identified risk factors for unwanted outcomes that need to be taken into consideration in clinical practice. Our model may contribute to earlier interventions for CKD and HF among patients with T2DM and ultimately improve the prognosis of these patients.

## Supplementary Information

Supplementary Information.

## Data Availability

The datasets generated and/or analyzed during the current study are available from the corresponding author on reasonable request.
