# Supplementary material for: Machine learning models for prediction of HF and CKD development in early-stage type 2 diabetes patients
Source: Sci Rep. 2022 Nov 21;12:20012. doi: 10.1038/s41598-022-24562-2 (PMC9678863; doi:10.1038/s41598-022-24562-2)
Supplement: Supplementary file 1 — Supplementary Information. [file 41598_2022_24562_MOESM1_ESM.docx]

# Supplementary Material

**Supplementary Table S1.** Japanese databases used in the present study.

|  | Medical Data Vision (MDV) | Real-world database (RWD) |
| --- | --- | --- |
| Data source | Claims and diagnosis procedure combination (DPC*) EMR data | Claims and DPC* EMR data |
| Number of facilities (as of 2020) | > 370 medical institutions | > 160 medical institutions |
| Number of patients (as of 2020) | > 20 million | Approximately 20 million |
| Data included | Patient characteristics, diagnoses, prescriptions, procedures, and laboratory test results for both inpatient and outpatient cares. | Patient characteristics, diagnoses, prescriptions, procedures, and laboratory test results for both inpatient and outpatient cares. |
| Remarks | MDV covers 22% of DPC hospitals (acute care hospitals) and is considered to have a large amount of these data compared with other databases. Laboratory test results from approximately 10% of the patient population, | Laboratory test results in almost all medical institutions. |
| Data period used in the present study | April 2008 to September 2018 | April 2008 to September 2020 |

*DPC is Japan’s own unique system of payment method for acute inpatient medical care. DPC covers diagnosis procedure combination per-diem payment system, a system by which provider reimbursement is calculated by a flat rate of per-diem fee based on the diagnosis group

## **Supplementary Table S2.** ATC codes, ICD-10 codes, and procedure codes for the inclusion and exclusion criteria.

| **Criteria** |  | **Code** |
| --- | --- | --- |
| **Inclusion** | T2DM | ICD 10: E11.x, E15.x |
|  | Antidiabetic medication | ATC code: A10 |
| **Exclusion** | T1DM | ICD 10: E10.x, O24.0 |
|  | Gestational diabetes | ICD10: O24.1, O24.4 |
|  | Stroke (including transient ischemic attack) | ICD10: I60.x, I61.x, I62.x, I63.x, I64.x, I65.x, I66.x, G45.x |
|  | MI | ICD10: I21.x, I22x, I25.2, I25.6 |
|  | Angina pectoris | ICD10: I20.1, I20.8, I20.9, I25.1  I25.5 |
|  | Unstable angina pectoris | ICD10: I20.0 |
|  | Atrial fibrillation | ICD10: I48.x |
|  | PAD | ICD10: I70.2, I73.9, I74.2, I74.3, I74.4, I74.5, I74.6, I74.7, I74.8, I74.9 |
|  | HF | ICD10: I50.x, I11.0, I13.0, I13.2 |
|  | Kidney disease | ICD10: N03.x, N05.x, N06.x, N07.x, N08.x, N17.x, N18.x, N19.x, E10.2, E11.2, E12.2, E13.2, E14.2, I12.X, I13.1, I13.2 |
|  | CABG | Procedure code: K551, K5511, K5512, K552, K5521, K5522, K5523, K552-21, K552-22 |
|  | PCI | Procedure code: K546, K5461, K5462, K5463, K547, K548, K5481, K5482, K549, K5491, K5492, K5493, K550, K550-2 |
|  | Cardiovascular revascularization therapy | Procedure code: K546, K5461, K5462, K5463, K547, K548, K5481, K5482, K549, K5491, K5492, K5493, K550, K550-2, K551, K5511, K5512, K552, K5521, K5522, K5523, K552-21, K552-22 |
|  | Intracarotid balloon pumping | Procedure code: K600, K6001, K6002 |
|  | Ventricular assist device | Procedure code: K603, K6031, K6032, K6033 |
|  | Implantation of pace maker | Procedure code: K597, K5971, K5972, K5973, K597-2, K597-3, K597-4, K598, K598-2 |
|  | Implantable cardioverter defibrillator | Procedure code: K599, K5991, K5992, K599-2, K599-3, K599-4, K599-5, K599-51, K599-52 |
|  | Percutaneous cardiopulmonary support | Procedure code: K602, K6021, K6022, K602-21, K602-22 |
|  | Percutaneous catheter ablation | Procedure code: K595, K5951, K5952, K5953, K595, K595-2 |
|  | Mitral valve replacement | Procedure code: K555, K5551, K5552, K5553, K5554, K555-2, K555-21, K555-22, K555-31, K555-32 |
|  | Mitral annuloplasty | Procedure code: K554, K5541, K5542, K5543, K554-21, K554-22 |
|  | Carotid stenting | Procedure code: K609-2 |
|  | Endarterectomy | Procedure code: K609, K6091, K6092, K6093, K609-2 |
|  | Surgical revascularization | Procedure code: K610-4 |
|  | Lower extremity amputation | Procedure code: K084, K0841, K0842, K084-2 |
|  | Dialysis | Procedure code: J38 |
|  | Kidney transplant | Procedure code: K780 |

ATC, Anatomical Therapeutic Chemical; CABG, coronary artery bypass graft; HF, heart failure; ICD-10, International Classification of Diseases, 10th Revision; MI, myocardial infarction; PAD, peripheral artery disease; PCI, percutaneous coronary intervention; T1DM, type 1 diabetes mellitus; T2DM, type 2 diabetes mellitus.

## **Supplementary Table S3.** ICD-10 codes and procedure codes for outcomes.

| **Outcome** | **Code** |
| --- | --- |
| HF | ICD10: I50.x, I11.0, I13.0, I13.2 |
| CKD | ICD10: N03.x, N05.x, N06.x, N07.x, N08.x, N17.x, N18.x, N19.x, E10.2, E11.2, E12.2, E13.2, E14.2, I12.x, I13.1, I13.2 |
| MI | ICD10: I21.x, I22.x |
| Stroke | ICD10: I60.x, I61.x, I62.x, I63.x |
| Dialysis | Procedure code: J38 |
| Kidney transplant | Procedure code: K780 |

CKD, chronic kidney disease; HF, heart failure; ICD-10, International Classification of Diseases, 10th Revision; MI, myocardial infarction.

**Supplementary Table S4.** Range of hyperparameters for the gradient boosting method.

| **Parameter name** | **Range of values to be explored** |
| --- | --- |
| Colsample_bytree | 0.4–0.85 |
| Gamma | 0.00001–0.1 |
| Lambda_l1 | 0–0.9 |
| Max_depth | 1–9 |
| Min_child_weight | 0–4.8 |
| Subsample | 0.6–0.9 |
| Learning_rate | 0.00001–0.01 |

**Supplementary Table S5.** Range of hyperparameters for the neural network method.

| **Parameter name** | **Range of values to be explored** |
| --- | --- |
| Number of intermediate layers | 1–4 |
| Number of parameters in the middle layer | 100–1,000 |
| Percentage of dropouts | 0–0.5 |
| Normalization method (Batch normalization) | None, yes |

**Supplementary Table S6.** AUROCs for the preliminary model.

|  | **Logistic regression** | | **Random forest analysis** | |
| --- | --- | --- | --- | --- |
| **Year** | **Diagnosis of CKD/HF** | **Hospitalization for CKD/HF** | **Diagnosis of CKD/HF** | **Hospitalization for CKD/HF** |
| 1 | 0.640 | 0.731 | 0.678 | 0.765 |
| 2 | 0.668 | 0.718 | 0.702 | 0.769 |
| 3 | 0.665 | 0.715 | 0.709 | 0.748 |
| 5 | 0.727 | 0.764 | 0.779 | 0,788 |

AUROC, area under the receiver operating characteristic curve; CKD, chronic kidney disease; HF, heart failure.

**Supplementary Table S7.** Predictive accuracy in XGB models with and without laboratory variables for the primary outcomes.

| **Diagnosis of CKD/HF** | | | | | **Hospitalization for CKD/HF** | | | | |
| --- | --- | --- | --- | --- | --- | --- | --- | --- | --- |
| **Predicted period**  **(year)** | **AUROC** | | | | **Predicted period**  **(year)** | **AUROC** | | | |
|  | **Learning** | **Verification** | | |  | **Learning** | **Verification** | | |
|  |  | **No laboratory value** | **Laboratory value present** | **Difference** |  |  | **No laboratory value** | **Laboratory value present** | **Difference** |
| 1 | 0.699 | 0.670 | 0.686 | 0.015 | 1 | 0.939 | 0.673 | 0.812 | 0.139 |
| 2 | 0.759 | 0.678 | 0.73 | 0.052 | 2 | 0.912 | 0.681 | 0.707 | 0.026 |
| 3 | 0.729 | 0.688 | 0.784 | 0.096 | 3 | 0.862 | 0.707 | 0.707 | 0.000 |
| 5 | 0.794 | 0.759 | 0.865 | 0.105 | 5 | 0.891 | 0.768 | 0.814 | 0.045 |

AUROC, area under the receiver operating characteristic curve; CKD, chronic kidney disease; HF, heart failure.

**Supplementary Table S8.** AUROC for the XGB model on the external validation set.

|  | **Year** | | | |
| --- | --- | --- | --- | --- |
| **Outcomes** | **1** | **2** | **3** | **5** |
| Diagnosis of CKD/HF | 0.644 | 0.633 | 0.618 | 0.718 |
| Hospitalization for CKD/HF | 0.733 | 0.725 | 0.775 | 0.837 |
| Diagnosis of CKD | 0.676 | 0.646 | 0.612 | 0.690 |
| Diagnosis of HF | 0.688 | 0.682 | 0.675 | 0.752 |
| HF hospitalization | 0.770 | 0.772 | 0.828 | 0.898 |
| Death | 0.906 | 0.898 | 0.878 | 0.869 |
| MACE composite | 0.672 | 0.650 | 0.667 | 0.743 |
| MARCE composite | 0.638 | 0.617 | 0.636 | 0.695 |

AUROC, area under the receiver operating characteristic curve; CKD, chronic kidney disease; HF, heart failure; MACE, major adverse cardiovascular event; MARCE, major adverse renal and cardiovascular events.

## **Supplementary Figure S1.** Methodology.


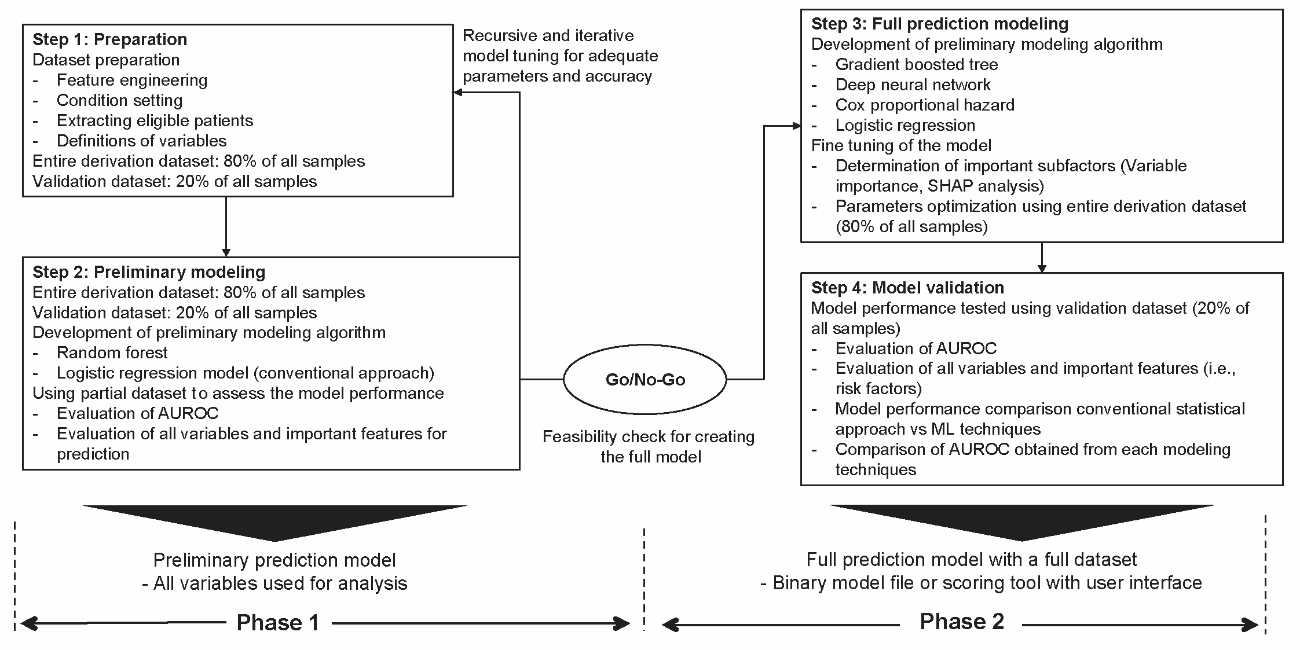
AUROC, area under the receiver operating characteristic curve; ML, machine learning; SHAP, SHapley Additive exPlanation.

**Supplementary Figure S2.** Laboratory variables employed for each outcome.


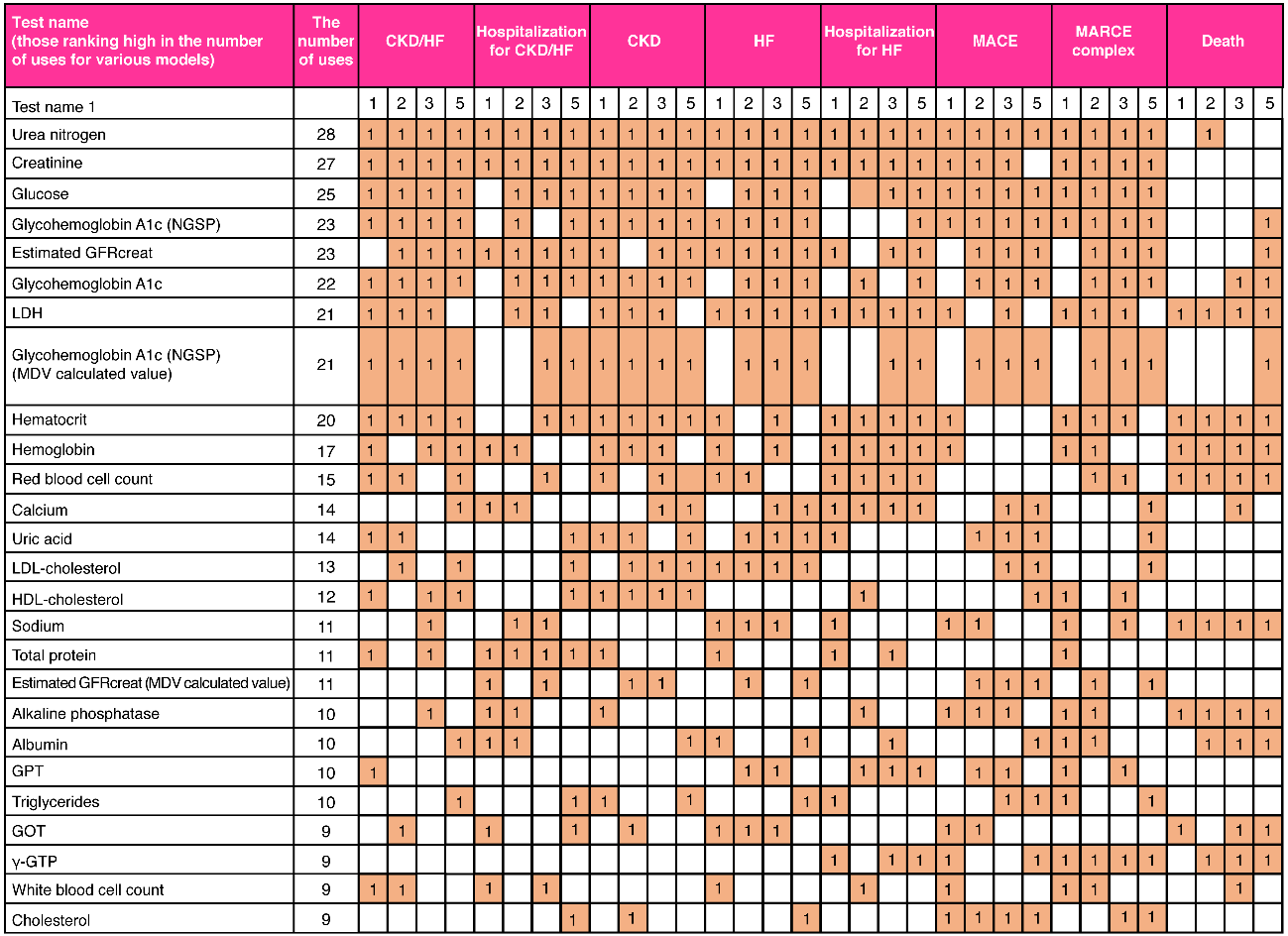
Variables with value 1 in the box (shaded area) were adopted; variables that are blank were not adopted.

CKD, chronic kidney disease; HF, heart failure; γ-GTP, γ-glutamyl transpeptidase; GFR, glomerular filtration rate; GOT, glutamic oxaloacetic transaminase; GPT, glutamic pyruvic transaminase; HDL-cholesterol, high-density lipoprotein cholesterol; LDH, lactate dehydrogenase; LDL-cholesterol, low-density lipoprotein cholesterol; MACE, major adverse cardiovascular event; MARCE, major adverse renal and cardiovascular events.

**Supplementary Figure S3.** Variable importance for laboratory values.


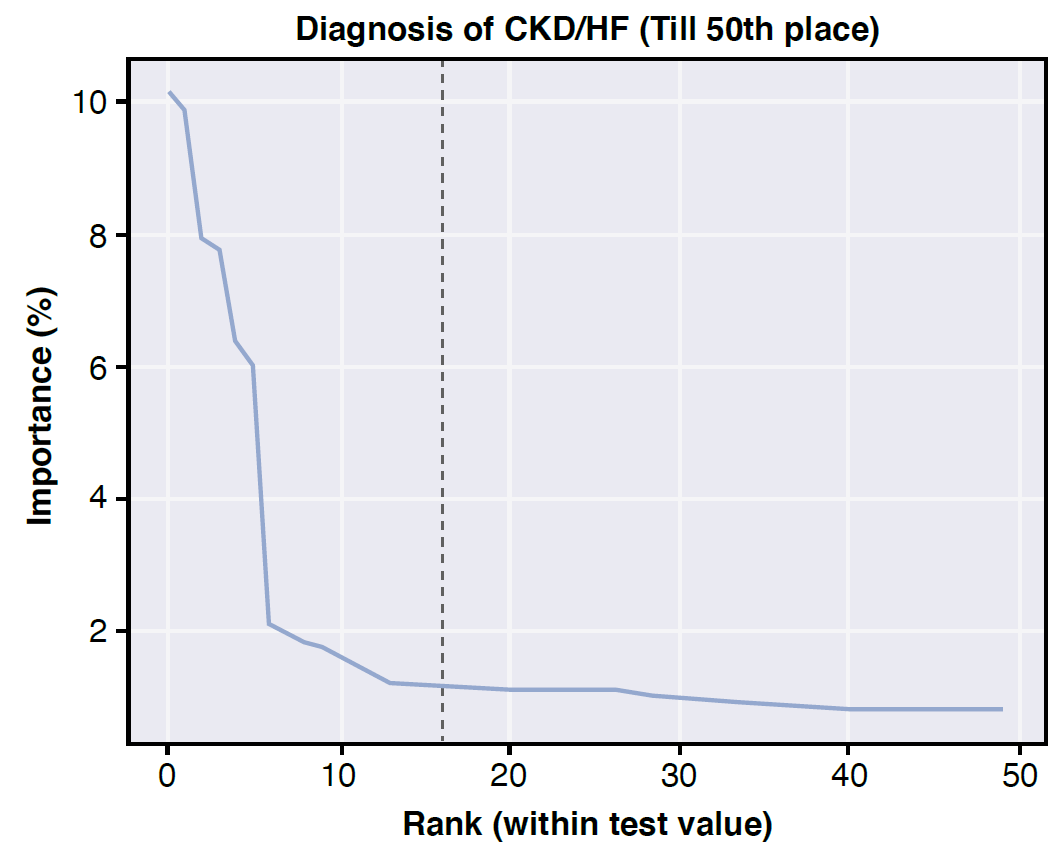


CKD, chronic kidney disease; HF, heart failure.

**Supplementary Figure S4.** Patient disposition in the external validation set.


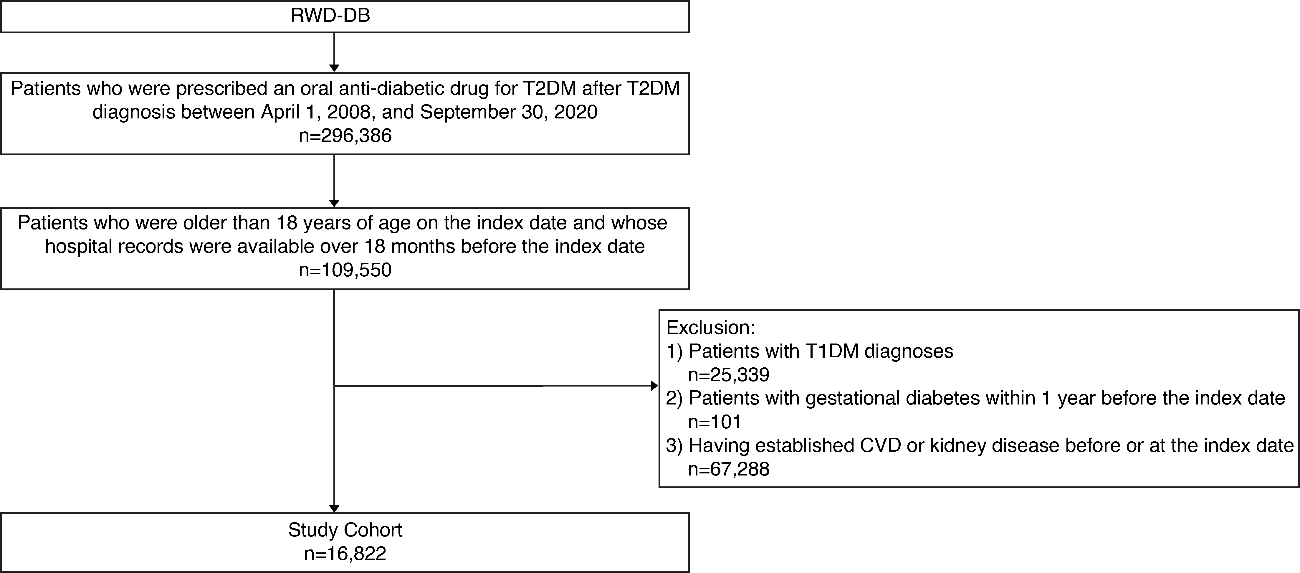


CVD, cardiovascular disease; RWD-DB, Real World Data database; T1DM, type 1 diabetes mellitus; T2DM, type 2 diabetes mellitus.

## **Supplementary Figure S5.** Variable importance of XGB tree including laboratory values for diagnosis of CKD/HF.


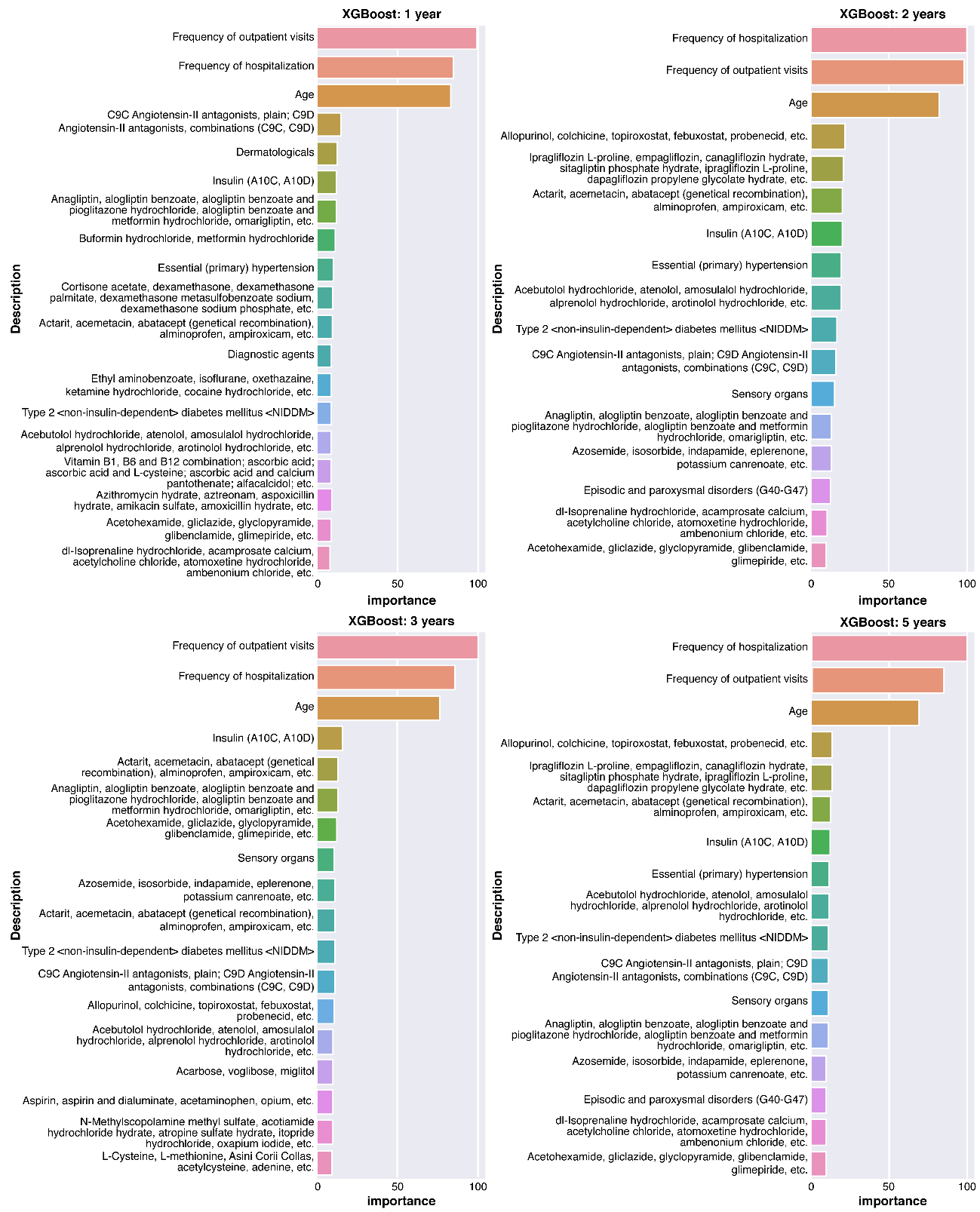


CKD, chronic kidney disease; HF, heart failure.

## **Supplementary Figure S6.** Variable importance of XGB tree including laboratory values for hospitalization due to CKD/HF.

##
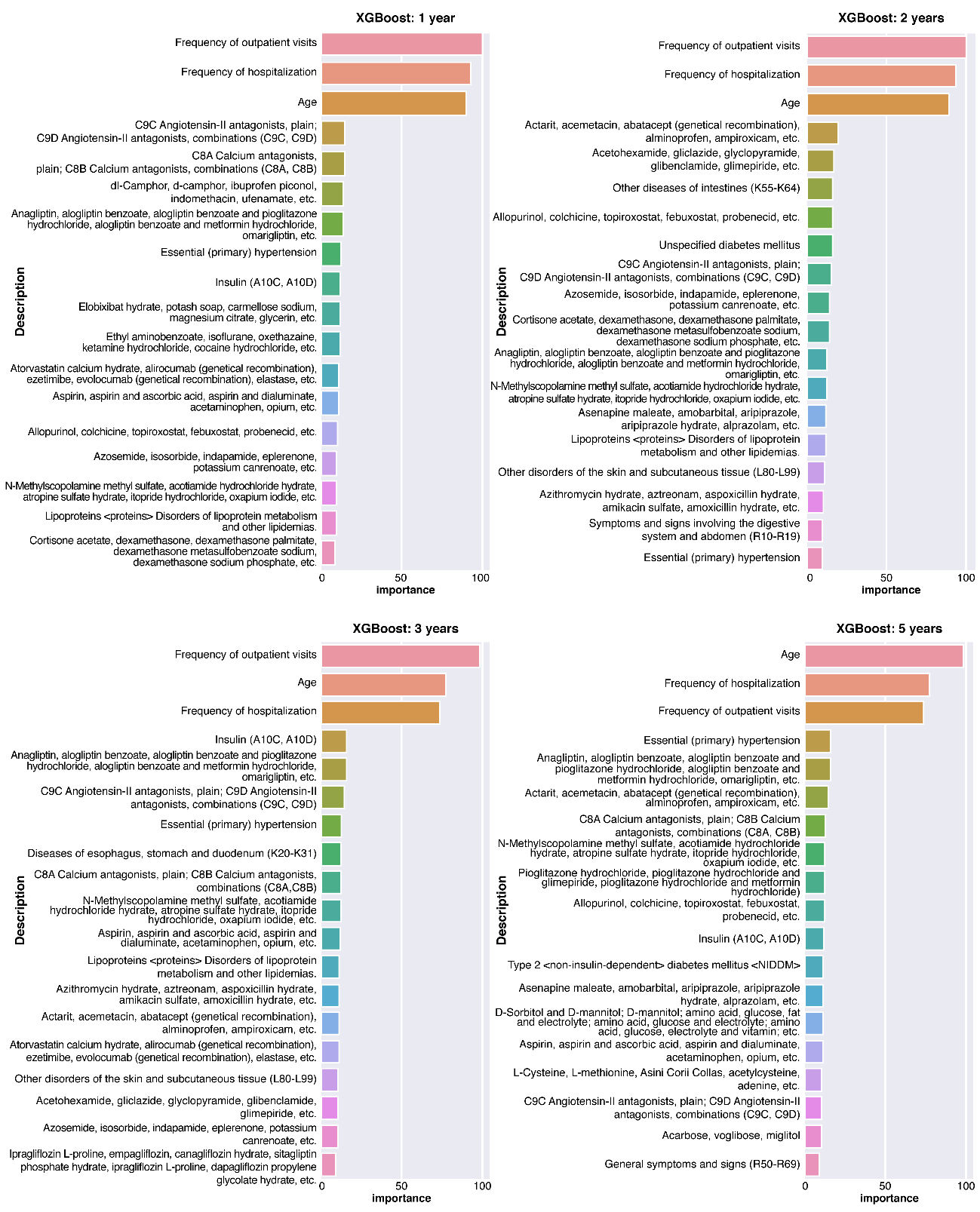


CKD, chronic kidney disease; HF, heart failure.

**Supplementary Figure S7.** 5-year Kaplan-Meier plots of high- and low-risk groups based on risk predictions for exploratory outcomes in the external validation set.


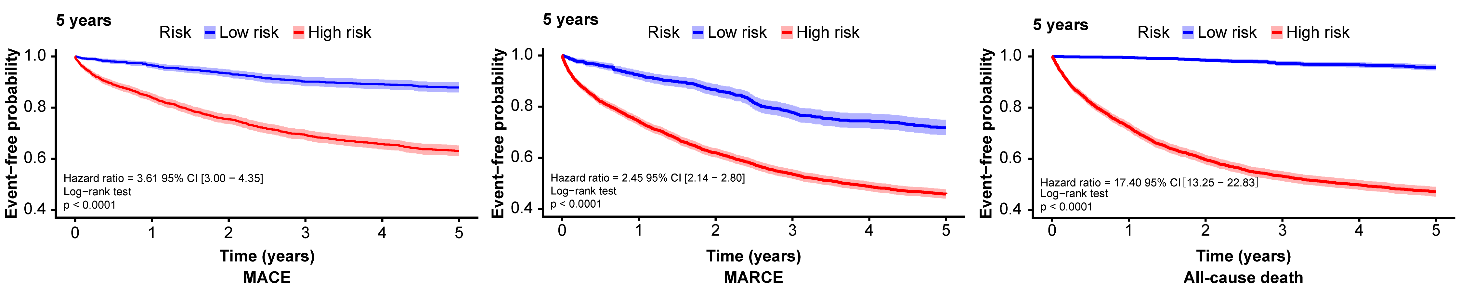


CI, confidence interval; MACE, major adverse cardiovascular event; MARCE, major adverse renal and cardiovascular events.
